# Long-Term Follow-Up of Prophylactic Mesh and Methodological Considerations

**DOI:** 10.3389/jaws.2025.14467

**Published:** 2025-06-11

**Authors:** Josep M. Garcia-Alamino, José Antonio Pereira Rodríguez, Manuel Lopez-Cano

**Affiliations:** ^1^ School of Health Sciences, Blanquerna Ramon Llull University, Barcelona, Spain; ^2^ Hospital del Mar, Parc de Salut Mar, Barcelona, Catalonia, Spain; ^3^ Vall d’Hebron University Hospital, Barcelona, Catalonia, Spain

**Keywords:** mesh complications, prophylactic mesh, methodology, long follow-up, mesh

Dear Editors,

The prevention of incisional hernias remains a significant challenge in surgical practice, particularly for high-risk patients undergoing midline laparotomies. Two recent studies exploring the long-term effectiveness and complications associated with prophylactic mesh placement (the PRIMA trial) offer valuable insights but also underscore critical methodological challenges that require attention.

The first study [1] evaluated mesh-related complications during subsequent relaparotomies, drawing on data from an observational follow-up of the PRIMA trial. While the study provided long-term data on mesh-related outcomes, its observational design introduced inherent limitations, including selection bias and unmeasured confounding factors. Data were only available from seven of the eleven participating centres, with operative notes missing for a substantial number of patients. This incomplete dataset risks creating an overly favourable impression of prophylactic mesh safety and efficacy. Additionally, the use of an “as-treated” rather than an “intention-to-treat” approach compounded potential biases further, as deviations from the protocol were not systematically accounted for. These methodological concerns highlight the necessity of employing tools such as ROBINS-I to assess the risk of bias in non-randomised studies, ensuring a balanced interpretation of findings [2]. The second study [3], which also analysed long-term data from the PRIMA trial, emphasised the superiority of prophylactic mesh placement over primary suture closure in reducing incisional hernia rates. However, this study faced significant challenges, including a high attrition rate and inconsistencies in follow-up protocols across centres. Of the 480 patients initially randomised, only 255 were available for extended follow-up, with data collected for just 142 patients. This substantial loss to follow-up raised questions about the representativeness of the cohort and introduced selection bias. Furthermore, while CT scans were reassessed by blinded radiologists to ensure objectivity, other outcome assessments relied on non-blinded methods, increasing the risk of subjective bias. The lack of strategies to address missing data, such as imputation or sensitivity analyses, further weakened the robustness of the conclusions.

Both studies underscore the critical role of methodological rigour in ensuring the reliability and applicability of research findings. Aside from the methodological details, another critical aspect is how data collection during follow-up is described. Ambiguous or overly technical descriptions can make it challenging for non-researcher surgeons to discern whether the data originate from a clinical trial or an observational study, potentially leading to misinterpretation of the findings. Clear and transparent reporting is essential to bridge this gap and support evidence-based decision-making in surgical practice.

Additionally, the studies highlight the need to contextualise findings within the broader framework of hernia biology [4]. The placement of prophylactic mesh, while effective in reducing hernia rates, may act as a delaying strategy rather than a definitive solution. Future research should therefore integrate biological considerations to better understand the multifactorial nature of hernia development and refine surgical interventions accordingly.

Moreover, these findings highlight the critical importance of surgeons possessing a robust understanding of scientific methodology. In the rapidly evolving field of surgical research, it is no longer sufficient to rely solely on technical expertise. Surgeons must be equipped with the skills to critically evaluate study designs, identify potential biases, and accurately interpret complex data. This methodological literacy is essential for distinguishing high-quality evidence from studies with significant limitations and ensuring that clinical decisions are informed by the most reliable and applicable research.

For example, a surgeon must understand the implications of selection bias or incomplete data management to assess whether a study’s findings can be generalised to their patient population. They should also recognise the importance of robust statistical techniques, such as sensitivity analyses or intention-to-treat approaches, in strengthening the validity of a study’s conclusions. Furthermore, familiarity with tools such as ROBINS-I empowers surgeons to systematically evaluate non-randomised studies and identify potential risks of bias that could compromise their clinical applicability.

In addition to these technical aspects, surgeons must also be aware of the broader context in which research findings are presented. The rise of predatory journals and the proliferation of poorly reviewed studies underscore the importance of critical appraisal skills for effectively navigating the growing body of surgical literature. Institutions and surgical societies play a crucial role in fostering a culture of critical enquiry by integrating research methodology training into surgical education and offering continuing professional development opportunities that focus on methodological literacy.

In conclusion, these studies contribute valuable data to the literature on incisional hernia prevention but also illustrate the complexities and limitations inherent in observational and long-term follow-up designs. To advance surgical research and practice, future studies must prioritise methodological rigour, including robust confounder adjustment, standardised follow-up protocols, and comprehensive methods for handling missing data. Moreover, fostering a culture of critical appraisal and methodological literacy among surgeons will be pivotal for navigating the evolving landscape of surgical research and ensuring the highest standards of patient care.

## Data Availability

The original contributions presented in the study are included in the article/supplementary material, further inquiries can be directed to the corresponding author.
